# Porcine Milk Oligosaccharides and Sialic Acid Concentrations Vary Throughout Lactation

**DOI:** 10.3389/fnut.2016.00039

**Published:** 2016-09-08

**Authors:** Austin T. Mudd, Jaime Salcedo, Lindsey S. Alexander, Stacey K. Johnson, Caitlyn M. Getty, Maciej Chichlowski, Brian M. Berg, Daniela Barile, Ryan N. Dilger

**Affiliations:** ^1^Piglet Nutrition and Cognition Laboratory, University of Illinois, Urbana, IL, USA; ^2^Department of Food Science and Technology, University of California Davis, Davis, CA, USA; ^3^Division of Nutritional Sciences, University of Illinois, Urbana, IL, USA; ^4^Mead Johnson Pediatric Nutrition Institute, Evansville, IN, USA; ^5^Foods for Health Institute, Food Science and Technology, University of California Davis, Davis, CA, USA; ^6^Department of Animal Sciences, University of Illinois, Urbana, IL, USA

**Keywords:** oligosaccharides, milk, porcine, sialic acid, milk composition

## Abstract

**Background:**

Milk oligosaccharides (OSs) are bioactive components known to influence neonatal development. These compounds have specific physiological functions acting as prebiotics, immune system modulators, and enhancing intestine and brain development.

**Objectives:**

The pig is a commonly used model for studying human nutrition, and there is interest in quantifying OS composition of porcine milk across lactation compared with human milk. In this study, we hypothesized that OS and sialic acid (SA) composition of porcine milk would be influenced by stage of lactation.

**Methods:**

Up to 250 mL of milk were collected from seven sows at each of three time points: day 0 (colostrum), days 7–9 (mature), and days 17–19 (weaning). Colostrum was collected within 6 h of farrowing and 3-day intervals were used for mature and weaning milk to ensure representative sampling. Milk samples were analyzed for OS profiles by Nano-LC Chip–QTOF MS, OS concentrations via HPAEC-PAD, and SA (total and free) was assessed by enzymatic reaction fluorescence detection.

**Results:**

Sixty unique OSs were identified in porcine milk. Neutral OSs were the most abundant at each lactation stage (69–81%), followed by acidic-sialylated OSs (16–29%) and neutral-fucosylated OSs (2–4%). As lactation progressed, acidic OSs decreased (*P* = 0.003), whereas neutral-fucosylated (*P* < 0.001) and neutral OSs (*P* = 0.003) increased throughout lactation. Six OSs were present in all samples analyzed across lactation [lacto-N-difucohexaose I (LNDFH-I), 2′-fucosyllactose (2′-FL), lacto-N-fucopentaose I (LNFP-I), lacto-N-neohexaose (LNnH), α1-3,β-4-d-galactotriose (3-Hex), 3′-sialyllactose (3′-SL)], while LDFT was present only in colostrum samples. Analysis of individual OS concentrations indicated differences (*P* = 0.023) between days 0 and 7. Conversely, between days 7 and 18, OS concentrations remained stable with only LNnH (*P* < 0.001) and LNDFH-I (*P* = 0.002) decreasing over this period. Analysis of free SA indicated a decrease (*P* < 0.001) as lactation progressed, while bound (*P* < 0.001) and total (*P* < 0.001) SA increased across lactation.

**Conclusion:**

Concentrations of OS differ between colostrum and mature milk in the pig, and SA concentrations shift from free to bound forms as lactation progresses. Our results suggest that although porcine milk OS concentration and the number of structures is lower than human milk, the OS profile appears to be closer to human milk rather than to bovine milk, based on previously published profiles.

## Introduction

The gastrointestinal tract is known to directly influence physiology of the entire organism. Relative to the rest of the body, the highest number of immune cells and a diverse microbiome are present in the gut. In the developing infant, gut maturation is influenced by oligosaccharides (OSs) present in the mother’s milk and OS composition of human milk changes throughout lactation, which likely has specific physiological implications in the developing infant. Piglets are considered a suitable model for human infant nutrition due to similarities in the development of intestinal physiology and nutrient requirements (1, 2). However, very little is known about the OS composition of porcine milk and the ways in which these concentrations change across lactation. Thus, there is interest in characterizing the bioactive components present in porcine milk that could modulate later gastrointestinal development, affording a meaningful comparison with human and other mammal’s milk composition. By understanding the similarities between porcine and human milk OS composition, researchers will be able to elucidate how specific OS aid in the development of the gastrointestinal tract.

Oligosaccharides are increasingly recognized as bioactive components of milk, and are believed to confer benefits throughout neonatal development (3). While the ratios of OS present in milk tend to vary among animal species, their presence exerts similar physiological effects, regardless of the animal species (4). Broadly speaking, functions of OS in neonatal development include prophylactically binding pathogens, acting as prebiotics for gut microbiota, modulation of intestinal and immune system development (5), and enhancing brain development (6, 7). Glycans present in human milk OS can serve as receptors for pathogens, such as rotavirus (8), *Escherichia coli, Vibrio cholera*, and *Salmonella fyris* (9), thereby inhibiting pathogen binding to mucosal surfaces (3). The prebiotic effects of OS promote colonization of beneficial Bifidobacteria in the neonate (5). As such, Bifidobacteria bind to intestinal epithelial cells, and the epithelial cells respond to inflammatory stimuli by releasing anti-inflammatory cytokines and decreasing release of inflammatory cytokines (10).

Sialic acid (SA)-containing structures are essential in the first stages of life for optimal development due to the biological processes in which they are involved: inhibition of pathogen binding, brain development, and immune system maturation (11–13). Sialic acid, both free and when incorporated into other compounds, such as sialyllactose (i.e., SA + lactose) plays an important role in immunological defenses of the young animal and lowers the risk of infection as gut maturation and microbial colonization occur (13, 14). Sialic acid is a main component of brain gangliosides that help in neural transmission and storage of information (15). Accordingly, SA supplementation in piglets has been shown to influence gene expression for neural cell adhesion molecules (16) and enhance learning and memory (7). Supplementation of sialyllactose increased ganglioside bound SA in the corpus callosum of piglets (17). Provided the multitude of ways in which SA supplementation influences infant development, there is interest in characterizing changes in free and bound SA in expressed milk throughout lactation.

While the functions of these molecules are clear, sensitive evaluation of the changes in OS profiles and concentrations throughout lactation is lacking. Due to their structural diversity in both the composing monosaccharides and their linkages, the identification and quantification of OS has proven difficult. Recent technological developments elevated mass spectrometry as one of the most valuable and widely used tools for characterization of OS in mammalian milks and other biological samples (18–20). Previous studies identified more than 100 OSs in human milk and 50 in bovine milk (18, 21, 22). To date, only a few studies have evaluated porcine milk OS, with up to 39 different OSs being characterized (23–25). The objective of our study was to quantify profiles of OS and SA in porcine milk throughout lactation to permit direct comparison with published profiles found in human milk. We hypothesized that day of lactation would impact the OS and SA concentrations of porcine milk, and we expand on recent research through identification of additional porcine milk OS as well as quantification of OS relative to known standards. Thus, findings from the present study may advance use of the porcine model for studying pediatric nutrition with outcomes, including how the gut microbiome, neurodevelopment, or immunomodulation are influenced by dietary OS.

## Materials and Methods

### Animals and Diets

Seven Yorkshire sows from the University of Illinois Imported Swine Research Laboratory (ISRL) were bred to Yorkshire boars and housed in standard gestation and farrowing crates throughout the study. Sows were provided custom gestation and lactation diets as described below. Two replicates of sows from consecutive farrowing groups were used with 3–4 sows in each replicate. Sows were allowed *ad libitum* access to water and were fed according to standard agricultural practices once each day (0700 h) during gestation and twice each day (0700 and 1600 h) during lactation to maintain body condition. Gestation diets were provided through 48 h post farrowing, at which time sows were provided lactation diets until 19 days of lactation. Corn and soy-protein isolate-based diets were formulated to meet requirements for all nutrients (Table 1) (26). A prophylactic antibiotic (BMD60, Alpharma, Bridgewater, NJ, USA) was added to sow diets according to manufacturer specifications starting on day 94 of gestation and continuing throughout lactation to prevent *Clostridium perfringens*-induced diarrhea in piglets. All animal care and experimental procedures were in accordance with the Guide for the Care and Use of Laboratory Animals and approved by the Institutional Animal Care and Use Committee of the University of Illinois.

**Table 1:** **Formulated and analyzed composition of gestation and lactation diets**.^a^

Ingredient, g/kg	Gestation	Lactation
Ground corn	785.0	652.0
Molassed dried sugar beet pulp	70.0	65.0
Soy protein isolate^b^	60.0	118.0
Cornstarch	39.7	101
Dicalcium phosphate	20.0	20.0
Corn oil	10.0	30.0
Limestone	7.5	6.5
Vitamin and mineral premix^c^	3.0	3.0
Choline chloride^d^	3.0	2.4
Bacitracin^e^	0.0	2.1
dl-Met	1.5	0.0
l-Trp	0.3	0.0
*Formulated composition, mg/kg*		
Choline	1,887	1,591
Folate	2.80	2.60
*Analyzed composition*		
Dry matter, %	90.6	88.8
***g/100 g of dry matter***		
Organic matter	95.6	95.6
Crude protein	13.7	18.3
Crude fat	2.69	2.58
Amino acids		
Lys	0.64	0.99
Met	0.42	0.25
Cys	0.23	0.25
Arg	0.75	1.11
Ile	0.54	0.77
Leu	1.42	1.57
Val	0.70	0.87
Phe	0.66	0.91
Thr	0.46	0.66
Trp	0.15	0.21

*^a^Experimental diets were formulated to meet or exceed nutrient and ME requirements for sows (26)*.

*^b^Ardex F, Archer Daniels Midland, Decatur, IL, USA*.

*^c^Provided per kilogram of complete diet: Ca, 318 mg (CaCO_3_); Zn, 125 mg (ZnSO_4_ H_2_O); Fe, 128 mg (FeSO_4_·H_2_O); Mn, 60 mg (MnSO_4_·H_2_O); Cu, 10.2 mg (CuSO_4_·5H_2_O); I, 1.3 mg (ethylenediamine dihydroiodide); Se, 0.3 mg (50% Se yeast, 50% Na_2_SeO_3_); vitamin A, 11,160 IU (retinyl acetate); vitamin D, 2,214 IU (cholecalciferol); vitamin E, 66 IU (DL-α-tocopherol acetate); vitamin K, 1.4 mg (menadione nicotinamide bisulfite); thiamine, 0.2 mg (thiamine mononitrate); riboflavin, 6.6 mg; niacin, 44 mg (nicotinamine); pantothenic acid, 24 mg (D-Ca pantothenate); pyridoxine, 0.2 mg (pyridoxine·HCl); biotin, 0.4 mg; folic acid, 1.6 mg; vitamin B_12_, 0.03 mg*.

*^d^Choline chloride 60%, Balchem, New Hampton, NY, USA*.

*^e^BMD60, Alpharma Inc., Bridgewater, NJ, USA*.

### Milk Collection

Milk samples were collected from each sow at three time-points throughout lactation. Colostrum was collected within 6 h of farrowing. To ensure representative samples of mature and weaning milk, samples were collected over 3-day collection periods, i.e., days 6–8 and days 17–19. Approximately 250 mL of milk were collected per sow at each time period. To facilitate milk let-down, piglets were removed from the sow for 1 h and placed in an adjacent, empty farrowing crate with access to supplemental heat and water. An intramuscular injection of oxytocin (2 mL, OxoJect, Henry Schein Animal Health, Dublin, OH, USA) was administered after 1 h without piglets, and milk was manually expressed into 50 mL conical tubes and stored at −20°C. Prior to analysis, milk samples were thawed, combined to create a homogeneous milk sample for each sow, and divided into aliquots; only one freeze–thaw process occurred prior to nutrient quantification procedures.

### Materials

Acetonitrile (ACN), formic acid (FA), trifluoroacetic acid (TFA), sodium hydroxide (NaOH), sodium chloride (NaCl), and sulfuric acid (H_2_SO_4_) were obtained from Thermo Fisher Scientific (Waltham, MA, USA); sodium acetate (NaAc) was purchased from Sigma-Aldrich (St Louis, MO, USA). All solvents were MS grade. OS standards for lacto-N-difucohexaose I (LNDFH-I), 2′-fucosyllactose (2′-FL), lacto-N-fucopentaose I (LNFP-I), lacto-N-tetraose (LNT), lacto-N-neotetraose (LNnT), lacto-N-neohexaose (LNnH), N-acetylgalactosaminyllactose, α1-3,β-4-d-galactotriose (3-Hex), 3′-sialyllactose (3′-SL), 6′-sialyllactose (6′-SL), and 6′-sialyl-N-acetyllactosamine (6′-SLN) were purchased from V-Labs Inc. (Covington, LA, USA), while LNH and LDFT standards were purchased from Prozyme Inc. (Hayward, CA, USA). The water used in all experiments was nanopure (18.2 ohms).

### Oligosaccharide Isolation and Purification

Oligosaccharides were isolated and purified according to a previously published method (27) with the following modifications: the crude OS extract was purified by solid-phase extraction using a Porous Graphitized Carbon microplate (PGC-SPE; Glygen Corp, Columbia, MD, USA), activated with three column volumes of 80% ACN, 0.1% TFA (v/v), and equilibrated with three column volumes of nanopure water. The OS-rich solution was loaded onto the cartridge, and salts were removed by washing with 10 column volumes of nanopure water. The OSs were then eluted with a solution of 40% ACN and 0.1% TFA (v/v) in water and dried in a speed vacuum centrifuge (miVac Quattro, Genevac, Ipswich, UK) at 35°C. Purified OSs were re-dissolved in 500 μL of nanopure water, sonicated for 10 min, and appropriately diluted for Nano-LC Chip–QTOF MS and HPAEC-PAD analyses.

### Oligosaccharides Profiling by Mass Spectrometry Nano-LC Chip–QTOF-MS

Nano-LC Chip–QTOF-MS/MS analysis was performed with an Agilent 6520 accurate-mass Quadrupole-Time-of-Flight (Q-TOF) LC/MS with a microfluidic nano-electrospray chip containing an enrichment and an analytical column packed with porous graphitized carbon (Agilent Technologies, Santa Clara, CA, USA). Nano-LC QTOF MS/MS parameters were as described previously (28) and a targeted porcine milk OS library was built using the OS identified by MS/MS; each sample was analyzed in triplicate.

Composition of eluted OS is listed as a set of the five individual monomers composing the OS using the following identification nomenclature: Hex_HexNAc_Fuc_Neu5Ac_Neu5Gc. Abbreviations for the components are as follows: Hex, hexose (glucose or galactose); HexNAc, N-acetylhexososamine; Fuc, fucose; Neu5Ac, N-acetylneuramic acid; Neu5Gc, N-glycolylneuramic acid. The number of each individual monomer present within an identified or quantified OS is represented using the aforementioned nomenclature. It should be noted that the analysis by Nano-LC Chip–QTOF MS separated multiple isomers for each OS; however, here we indicate the relative percentage of composition 3_1_0_0_0 (3 Hex, 1 HexNAc) as the sum of LNT and LNnT. Similarly, the relative percentage of composition 4_2_0_0_0 (4 Hex, 2 HexNAc) was the sum of LNH and LNnH due to close eluting times of the isomer pairs (i.e., co-elution).

### Oligosaccharide Quantification by HPAEC-PAD

Quantification of eight neutral (LNDFH, LDFT, 2′-FL, LNFP-I, LNT, LNnT, N-acetylgalactosaminyllactose, and 3-Hex) and 3 acidic (6′-SLN, 6′-SL, and 3′-SL) OSs was carried out using high-performance anion-exchange chromatography with pulsed amperometric detection (HPAE-PAD ICS-5000; Thermo Fisher Scientific, Waltham, MA, USA). Diluted OS solutions were filtered through a 0.22 μm membrane and injected using a 25 μL loop. Chromatographic separation was carried out on a CarboPac PA200 analytical column (3 mm × 250 mm; Dionex, Sunnyvale, CA, USA) and a CarboPac PA200 guard column (3 mm × 50 mm; Dionex, Sunnyvale, CA, USA) with 0.5 mL/min elution and a non-isocratic gradient: 0–10 min 50% B, 10–50 min 45% B – 10% C. The column was equilibrated for 5 min with 10% B followed by 10 min with 50% B. Solvent A was deionized water, solvent B 200 mM NaOH, and solvent C was 100 mM NaAc in 100 mM NaOH. Quantification was assessed by external calibration using a mixture of all OS standards ranging from 0.0001 to 0.03 g/L.

### Quantification of Sialic Acid by Enzymatic Reaction and Fluorescence Detection

To evaluate total SA content, an acid hydrolysis was performed: 200 μL of sample was mixed with 800 μL of 0.05 M H_2_SO_4_, heated (60 min at 80°C), cooled to room temperature and centrifuged (13,000 × *g* for 30 min at 4°C). For free SA determination, 1 mL of sample was centrifuged (13,000 × *g* for 30 min at 4°C), the aqueous phase was collected, and 400 μL of the supernatant was purified by solid phase extraction using strong anionic exchange cartridges (OnGuard II-A; Dionex, Sunnyvale, CA, USA) that had been previously activated with 10 mL deionized water. After sample loading, each cartridge was washed with 10 mL of distilled water and SA was eluted with 10 mL of 100 mM NaCl. Dried samples were reconstituted in 500 μL of deionized water, vortexed, and sonicated to assure complete solubilization. SA (expressed as Neu5AC) concentrations were assessed to determine free and total SA in each sample using an enzymatic commercial kit with fluorescence detection (ab83375; Abcam, Cambridge, UK) as described by the manufacturer. Subsequently, the quantity of bound SA was calculated as the difference between total and free SA concentrations.

### Statistical Analysis

A one-way repeated measures ANOVA was performed using the MIXED procedure of SAS 9.3 (SAS Institute, Cary, NC, USA) to evaluate differences in the relative proportion of OS and individual concentrations of OS and SA in porcine milk throughout lactation. Day of lactation served as the repeated measure, and an HSD-Tukey adjustment was applied during the *post hoc* data analysis. Sow served as the experimental unit for all analyses. Prior to statistical analysis, normality and homoscedasticity of the data were checked using the Kolmogorov–Smirnov and Levene tests, respectively; all data were normally distributed and no outliers were identified. Data are presented as least squares means for each lactation time-point. Statistical significance was considered when *P* < 0.05.

## Results

### Oligosaccharide Profiling by Mass Spectrometry Nano-LC Chip–QTOF MS

Sixty OSs (including isomers and anomers) were identified and confirmed by Nano-LC Chip–Q-TOF MS/MS in this study (Table 2). Six OSs comprised 60% of the total (2 Hex-1 Neu5Ac or sialyllactose; 3 Hex-1 HexNAc; 3 Hex; 4 Hex-1 HexNAc; 4 Hex-2 HexNAc; and 4 Hex-2 HexNAc-1 Neu5Ac), with 4 Hex-2 HexNAc (25–33%) being the most abundant at each stage of lactation. 3′-Sialyllactose was the second most abundant in colostrum, accounting for 8% of the total OS.

**Table 2:** **Qualitative profiles and relative abundances of oligosaccharides (OS) in porcine milk throughout lactation**.

#	Composition^1^	Formula	Exact mass(exact)	Retention Time, min	Type^2^	Relative abundance (%) by Day of Lactation^3^			±SEM	*P*-value	Presence in human milk^4^
						0	7	18			
1	2_0_1_0_0	C18 H32 O15	488.1738	12.11	NF	*0.33^a^*	2.37^b^	1.87^b^	0.270	**<0.0001**	✓
2	2_0_1_0_0	C18 H32 O15	488.1723	14.37	NF	0.40	0.36	0.41	0.055	0.5507	✗
3	2_0_1_0_0	C18 H32 O15	488.1737	15.62	NF	0.71	0.71	0.74	0.106	0.9579	✗
4	3_0_0_0_0	C18 H32 O16	504.1694	12.40	N	4.31^a^	1.43^b^	*1.07^b^*	0.559	**0.0011**	✗
5	3_0_0_0_0	C18 H32 O16	504.1690	13.02	N	3.55^a^	1.91^b^	2.03^b^	0.366	**<0.0001**	✗
6	3_0_0_0_0	C18 H32 O16	504.1690	13.38	N	6.54^a^	2.74^b^	2.90^b^	0.671	**0.0007**	✗
7	3_0_0_0_0	C18 H32 O16	504.1692	14.41	N	2.48^a^	2.06^b^	2.05^b^	0.247	**0.0299**	✗
8	3_0_0_0_0	C18 H32 O16	504.1696	15.66	N	0.58^a^	0.80^a^	1.54^b^	0.212	**0.0002**	✗
9	3_0_0_0_0	C18 H32 O16	504.1688	16.45	N	0.30^a^	0.27^a^	0.51^b^	0.082	**0.0200**	✗
10	1_0_0_1_0	C18 H32 O15	470.1508	15.78	A	*0.01*	*0.07*	*0.22*	0.131	0.5029	✓
11	2_0_0_1_0	C23 H39 N O19	633.2118	18.30	A	1.65	1.09	1.60	0.273	0.3404	✓
12	2_0_0_1_0	C23 H39 N O19	633.2117	23.37	A	9.73	5.98	4.76	1.610	0.0816	✓
13	1_1_0_1_0	C25 H42 N2 O19	674.2374	16.77	A	0.25^a^	*0.05^b^*	*0.04^b^*	0.025	**<0.0001**	✗
14	1_1_0_1_0	C25 H42 N2 O19	674.2377	18.31	A	1.16^a^	*0.21^b^*	*0.14^b^*	0.105	**<0.0001**	✓
15	1_1_0_1_0	C25 H42 N2 O19	674.2368	22.94	A	0.29^a^	*0.01^b^*	*0.00^b^*	0.028	**<0.0001**	✓
16	3_1_0_0_0	C26 H45 N O21	707.2477	15.23	N	1.07^a^	0.69^b^	0.50^b^	0.087	**0.0007**	✓
17	3_1_0_0_0	C26 H45 N O21	707.2478	16.00	N	0.99	*0.53*	0.88	0.307	0.5746	✓
18	3_1_0_0_0	C26 H45 N O21	707.2484	17.14	N	6.89	4.90	5.68	1.404	0.6366	✗
19	3_1_0_0_0	C26 H45 N O21	707.2477	21.64	N	0.90^a^	0.97^a^	0.56^b^	0.102	**0.0236**	✗
20	2_2_0_0_0	C28 H48 N2 O21	748.2742	15.28	N	0.65^a^	0.33^b^	0.26^b^	0.082	**0.0034**	✗
21	3_0_0_1_0	C29 H49 N O24	795.2641	24.11	A	0.03	0.29	0.45	0.162	0.2025	✗
22	3_0_0_1_0	C29 H49 N O24	795.2641	25.19	A	*0.35^a^*	0.77^ab^	1.06^b^	0.182	**0.0335**	✗
23	3_0_0_1_0	C29 H49 N O24	795.2644	25.80	A	0.39^a^	0.63^ab^	0.87^b^	0.099	**0.0051**	✗
24	4_1_0_0_0	C32 H55 N O26	869.3013	19.69	N	5.36	7.37	7.11	0.672	0.1088	✗
25	4_1_0_0_0	C32 H55 N O26	869.3012	20.70	N	5.43	7.69	7.44	1.076	0.2922	✗
26	4_1_0_0_0	C32 H55 N O26	869.3005	27.54	N	1.16	2.14	1.93	0.384	0.1309	✗
27	3_2_0_0_0	C34 H58 N2 O26	910.3274	18.51	N	*0.28*	*0.22*	0.65	0.249	0.4526	✗
28	3_2_0_0_0	C34 H58 N2 O26	910.3288	19.08	N	*0.56*	1.05	1.06	0.361	0.5082	✗
29	3_2_0_0_0	C34 H58 N2 O26	910.3286	19.39	N	*1.05*	*1.11*	1.58	0.680	0.8237	✗
30	3_1_0_1_0	C37 H62 N2 O29	998.3431	24.73	A	0.36	*0.09*	0.30	0.129	0.2926	✓
31	3_1_0_1_0	C37 H62 N2 O29	998.3457	28.69	A	1.73^a^	0.31^b^	*0.06^b^*	0.131	**<0.0001**	✓
32	3_1_0_1_0	C37 H62 N2 O29	998.3448	26.35	A	0.10	0.11	0.07	0.025	0.3424	✓
33	4_1_1_0_0	C38 H65 N O30	1015.3573	15.38	NF	0.10^a^	*0.02^b^*	*0.00^b^*	0.024	**0.0106**	✗
34	4_2_0_0_0	C40 H68 N2 O31	1072.3836	21.64	N	18.71^a^	30.75^ab^	29.66^b^	3.038	**0.0179**	✗
35	4_1_0_1_0	C43 H72 N2 O34	1160.3979	27.14	A	*0.29^a^*	1.12^b^	0.70^b^	0.163	**0.0005**	✗
36	4_1_0_1_0	C43 H72 N2 O34	1160.3982	27.54	A	0.78^a^	2.04^b^	1.15^a^	0.340	**0.0009**	✗
37	4_2_0_1_0	C51 H85 N3 O39	1363.4791	26.13	A	7.03	6.19	*2.73*	1.324	0.0704	✓
38	4_2_0_1_0	C51 H85 N3 O39	1363.4783	28.93	A	3.12^a^	*0.12^b^*	*0.03^b^*	0.286	**<0.0001**	✓
39	1_0_0_1_0	C17 H29 N O14	471.1616	11.70	A	*0.01^a^*	0.14^ab^	0.21^b^	0.043	**0.0149**	✓
40	1_1_1_0_0	C20 H35 N O15	529.1994	13.18	NF	0.15^a^	0.13^a^	0.06^b^	0.025	**0.0158**	✗
41	1_1_1_0_0	C20 H35 N O15	529.1993	13.73	NF	0.12^a^	0.05^b^	*0.03^b^*	0.022	**0.011**	✗
42	2_1_0_0_0	C20 H35 N O16	545.1953	11.35	N	*0.55^a^*	1.02^ab^	1.94^b^	0.335	**0.0054**	✓
43	2_1_0_0_0	C20 H35 N O16	545.1954	12.05	N	0.60^a^	1.48^ab^	2.55^b^	0.590	**0.0052**	✓
44	2_1_0_0_0	C20 H35 N O16	545.1954	12.98	N	*1.46^a^*	3.31^b^	4.66^b^	0.482	**0.0004**	✓
45	2_1_0_0_0	C20 H35 N O16	545.1953	13.96	N	*0.35^a^*	0.77^ab^	1.88^b^	0.320	**0.0102**	✓
46	2_1_0_0_0	C20 H35 N O16	545.1953	14.38	N	0.30^a^	0.77^b^	0.72^ab^	0.226	**0.0290**	✓
47	2_1_0_0_0	C20 H35 N O16	545.1951	15.70	N	*0.12*	0.21	0.34	0.099	0.2928	✓
48	4_0_0_0_0	C24 H42 O21	666.2211	2.42	N	0.28^a^	0.33^ab^	0.75^b^	0.109	**0.0147**	✗
49	4_0_0_0_0	C24 H42 O21	666.2215	12.72	N	0.16	0.18	0.10	0.044	0.338	✗
50	4_0_0_0_0	C24 H42 O21	666.2212	19.93	N	0.16	0.11	0.09	0.023	0.065	✗
51	2_1_1_0_0	C26 H45 N O20	691.2525	12.97	NF	*0.00^a^*	0.18^b^	0.10^ab^	0.034	**0.0048**	✗
52	2_1_0_1_0	C31 H52 N2 O24	836.2902	13.79	A	*0.01*	*0.01*	*0.18*	0.108	0.4052	✓
53	2_1_0_1_0	C31 H52 N2 O24	836.2894	14.96	A	1.02	*0.11*	*0.65*	0.409	0.2439	✓
54	2_1_0_1_0	C31 H52 N2 O24	836.2899	22.71	A	*0.11*	*0.10*	0.21	0.078	0.5575	✓
55	3_1_1_0_0	C32 H55 N O25	853.3067	15.80	NF	0.35^a^	0.06^b^	0.07^b^	0.054	**0.0018**	✓
56	5_1_0_0_0	C38 H65 N O31	1031.3523	22.80	N	0.15	0.07	*0.04*	0.031	0.0571	✗
57	3_3_0_0_0	C42 H71 N3 O31	1113.4093	22.32	N	1.15^a^	*0.08^b^*	*0.02^b^*	0.130	**<0.0001**	✗
58	3_3_0_0_0	C42 H71 N3 O31	1113.4092	20.12	N	3.13^a^	1.18^b^	0.67^b^	0.392	**0.0009**	✗
59	7_0_0_0_0	C42 H72 O36	1152.3789	17.83	N	*0.01*	0.02	*0.01*	0.007	0.4861	✗
60	3_2_0_1_0	C45 H75 N3 O34	1201.4255	25.60	A	0.18	0.17	*0.10*	0.061	0.4814	✗

*^1^Composition (in order): Hex_HexNAc_Fuc_Neu5Ac_Neu5Gc. Hex, hexose (glucose or galactose); HexNAc, N-acetylgalactosamine; Fuc, fucose; Neu5Ac, N-acetylneuraminic acid; Neu5Gc, N-glycolylneuraminic acid*.

*^2^Type of oligosaccharide: N, neutral OS; NF, neutral-fucosylated OS; A, acidic OS*.

*^3^Relative abundance expressed as a mean of seven different porcine milk samples for each time-point analyzed in triplicate. Data expressed relative to the total amount of oligosaccharides detected in each individual porcine milk sample. Italicized means are not statistically different than 0 (*P* < 0.05). ^ab^Means without a common superscript letter within a row differ (*P* < 0.05)*.

*^4^Qualitative profile comparison between porcine and human milk oligosaccharides (21, 22)*.

**Figure 1:**
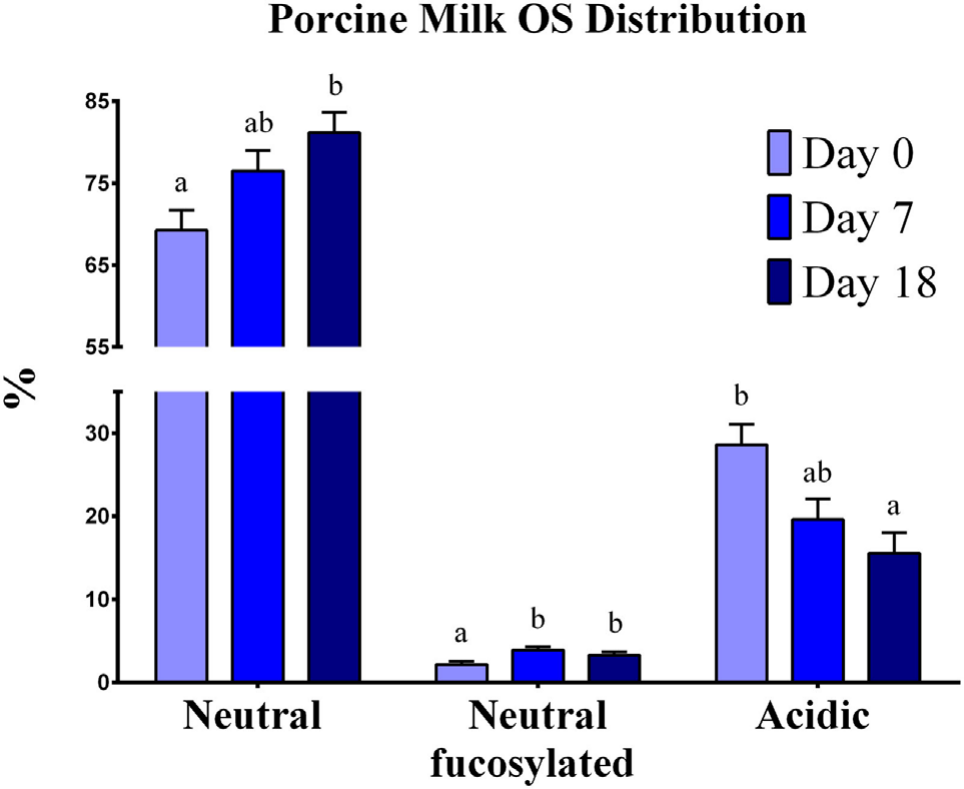
**Oligosaccharide distribution in porcine milk**. Results are expressed as the average of the relative abundance (%) of OS found in porcine milk from seven sows throughout the lactation period. Neutral, neutral-fucosylated, and acidic OS distributions exhibited changes by day (*P* < 0.05) throughout lactation. ^a,b^Indicate statistical differences among time of milk collection during the lactation period, where means that do not share a common superscript letter differ (*P* < 0.05, HSD-Tukey Test).

Mature milk contained mainly neutral non-fucosylated OS, 4 Hex-2 HexNAc, and 3-Hex comprising more than 35% of the total OS. Neutral OSs were the most abundant at each lactation stage (69–81%), followed by acidic-sialylated OS (16–29%) and neutral-fucosylated OS (2–4%) (Figure 1). As lactation progressed, a shift in the type of OS abundances was apparent, exhibited as a decrease (*P* = 0.0026) in acidic OS and an increase in neutral-fucosylated (*P* < 0.0001) and neutral (*P* = 0.0031) OS from the beginning to the end of lactation.

### Oligosaccharide Absolute Quantification by HPAEC-PAD

**Figure 2:**
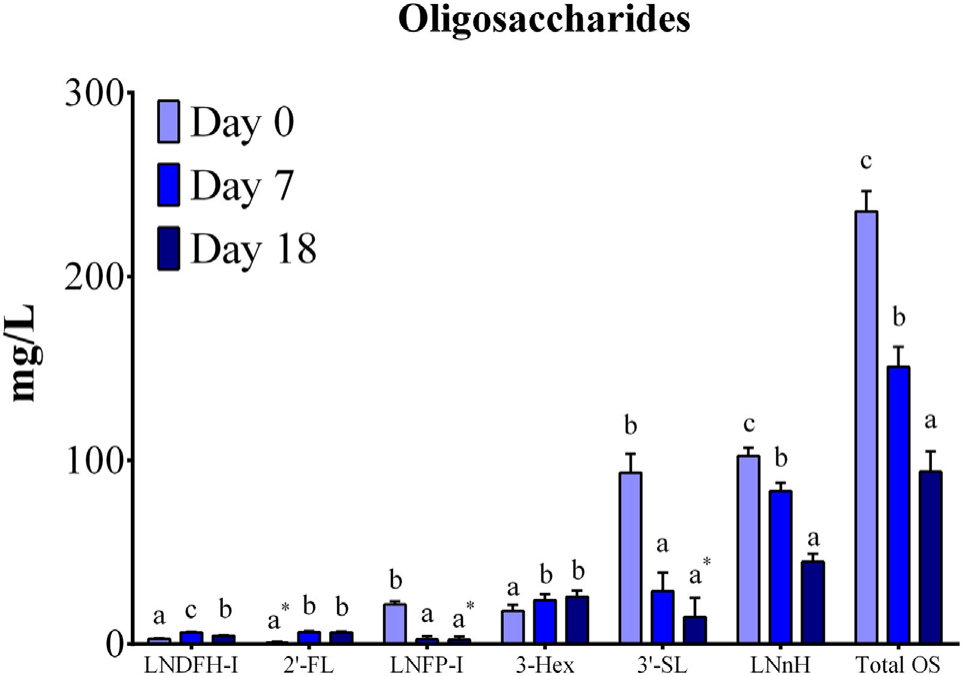
**Oligosaccharide quantification by HPAE-PAD (expressed as mg/L) in porcine milk**. Total OS concentrations decreased (*P* < 0.0001) across lactation. Individual differences were observed for six quantified OS, with all OS exhibiting changes in concentration (*P* < 0.05) throughout lactation. The following OS had concentrations below detectable limits for the specified number of sows (*n*) for at least one analyzed time point: 2′FL (*n* = 4), LNFP-I (*n* = 5), 3-Hex (*n* = 1), 3-SL (*n* = 1). Means with an * are not statistically different than 0. ^abc^Indicate statistical differences among time of milk collection during the lactation period, where means that do not share a common superscript letter differ (*P* < 0.05, HSD-Tukey Test). Abbreviations: LNDFH-I, lacto-N-difucohexaose I; 2′-FL, 2′-fucosyllactose; LNFP-I, lacto-N-fuctopentaose I; 3-Hex, α1-3,β1-4-d-galactotriose; 3′-SL, 3′-sialyllactose; LNnH, lacto-N-neohexaose.

Eight neutral OS (LNDFH-I, LDFT, 2′-FL, LNFP-I, LNT, LNnH, 2Hex-1HexNAc, and 3 Hex) and three acidic (6′-SLN, 6′-SL, and 3′-SL) were quantified using reference standards. Six OSs were present in all the samples analyzed across lactation (LDFH-I, 2′-FL, LNFP-I, LNnH, 3 Hex, 3′-SL; Figure 2), while LDFT was present only in colostrum samples (data not shown). The OS LNT, 2Hex-1HexNAc, 6′-SLN, and 6′-SL were not quantifiable in any sample due to concentrations being below detectable levels.

Total quantified OS concentrations in porcine milk decreased (*P* < 0.0001) throughout lactation (Figure 2). Differences across lactation were also observed for LNDFH-I (*P* < 0.0001), 2′-FL (*P* < 0.0001), LNFP-I (*P* < 0.0001), 3-Hex (*P* = 0.0157), 3′-SL (*P* < 0.0001), and LNnH (*P* < 0.0001). Further analysis indicated decreases (*P* < 0.001) in LNFP-I concentrations at each lactation point. Both 2′FL and 3-Hex increased (*P* < 0.05) from days 0 to 7 but remained constant from days 7 to 18. Conversely, LNFP-I and 3′-SL indicated decreases (*P* < 0.001) from days 0 to 7 while remaining stable from days 7 to 18. A decrease in concentration was observed for LNnH (*P* < 0.001) and LNDFH-I (*P* = 0.002) from days 7 to 18. These results match relative abundance findings in the OS profiling, where sialylated OS decreased throughout lactation and fucosylated OS showed a small, but significant, increase. In general, the most abundant OSs were LNnH (~50 mg/L) and 3′-SL (~30 mg/L), followed by 3-Hex (~20 mg/L), when averaged over lactation.

### Total and Free Sialic Acid quantification by Enzymatic Reaction – Fluorescence Detection

**Figure 3:**
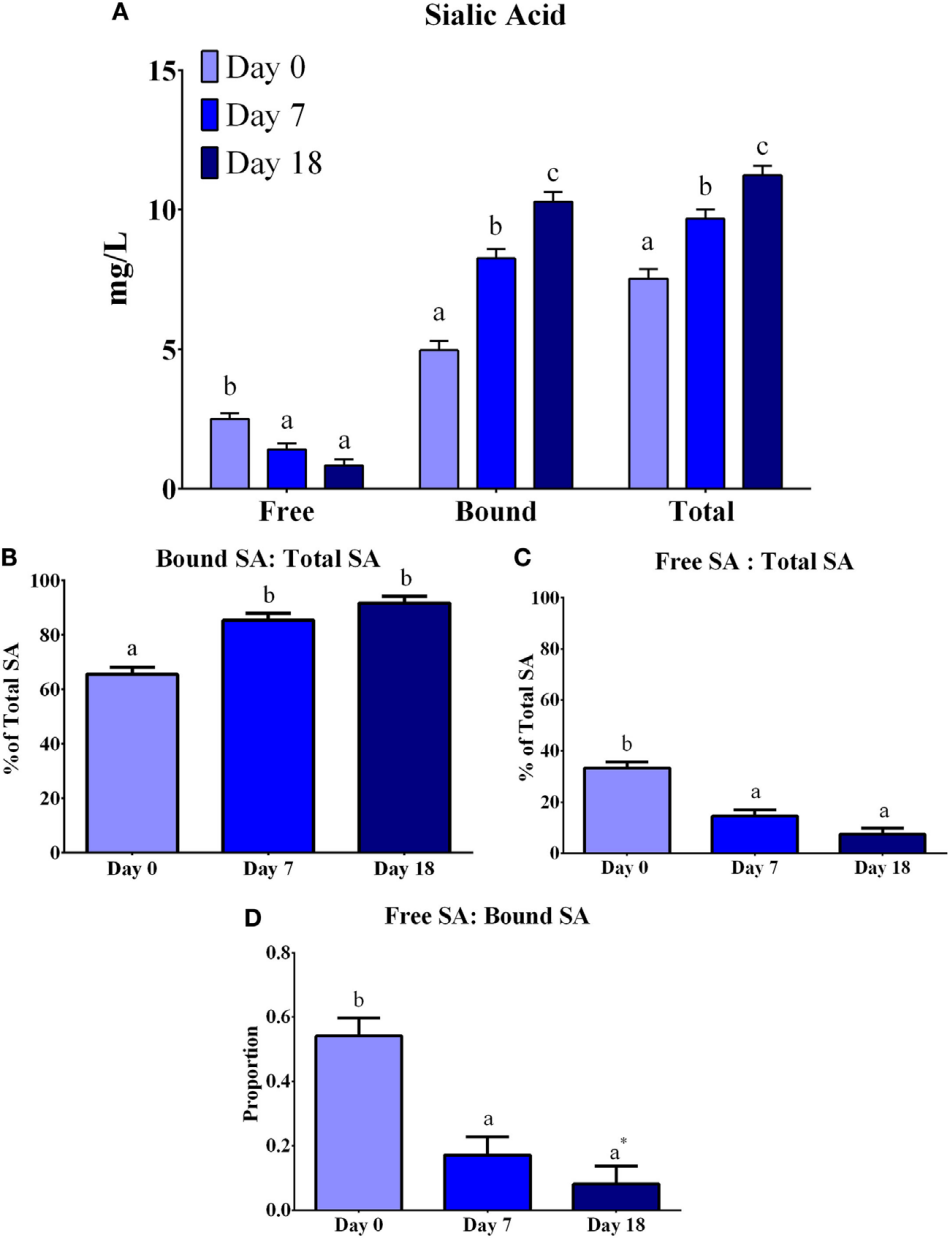
**Total, free, and bound sialic acid (SA) concentration (expressed as mg NeuAc/L) in porcine milk**. Bound SA was determined from the difference between total and free SA. **(A)** Concentrations of free SA decreased (*P* < 0.0001), whereas bound and total SA increased (*P* < 0.0001) across lactation. **(B)** An increase (*P* < 0.0001) was observed for the ratio of bound-to-free SA from days 0 to 7 and remained constant after day 7. **(C)** The ratio of free-to-total SA decreased (*P* < 0.0001) from days 0 to 7 and then remained constant from days 7 to 18. **(D)** The ratio of free-to-bound SA decreased (*P* < 0.0001) from days 0 to 7 and remained constant from days 7 to 18. Means with an asterisk (*) are not statistically different than 0 (*P* < 0.05). ^abc^Indicate statistical differences among time of milk collection during the lactation period, where means that do not share a common superscript letter differ (*P* < 0.05, HSD-Tukey Test).

The concentrations of total, free, and bound SA were determined using an enzymatic reaction with fluorescence detection (Figure 3). Overall, differences (*P* < 0.0001) were observed between lactation time-points for each of free, bound, and total SA. Free SA concentrations decreased (*P* = 0.0126) from days 0 to 7, but remained constant from days 7 to 18, whereas bound and total SA concentrations increased (*P* < 0.0001) at each lactation time-point. The ratio of free-to-total SA decreased (*P* < 0.0001) from days 0 to 7, but this ratio did not change from days 7 to 18. Conversely, the ratio of bound-to-total SA increased (*P* < 0.0001) from days 0 to 7, but remained constant from days 7 to 18. The ratio of free-to-bound SA indicated a decrease (*P* < 0.0001) across lactation, where the proportion of free-to-bound SA decreased (*P* = 0.0012) from days 0 to 7 and remained constant from days 7 to 18.

## Discussion

Oligosaccharides are a class of molecules that have gained considerable attention because of the biological processes in which they impact the first stages of life (5). Because the piglet is a widely used model for human infant nutrition, it is necessary to understand how the OS composition of porcine milk compares to human milk. By characterizing these similarities and the changes that occur across lactation, researchers will be able to use this knowledge to aid in development of future gut development studies. In the present study, porcine milk was analyzed to identify the OS variation during lactation and, when availability of known standards allowed, quantify the most abundant OS compounds. Additionally, free, bound, and total SA concentrations were analyzed to quantify concentrations and relative changes in porcine milk throughout lactation.

Sixty OSs (including isomers and anomers) were identified in porcine milk during the first 18 days of lactation by Nano-LC Chip–Q-TOF MS, with 24 structures matching those identified in human milk (21, 22). The total number of OSs identified is within the range described previously (23, 25), though a number of novel isoforms were identified in this study, suggesting potential diversity due to genetic or environmental influences. However, the relative amount of neutral OS identified in the present study (73–78%) is higher than what was previously determined for porcine colostrum (20%) (23, 25). Also, the percentage of fucose-containing OS increased as lactation progressed, while sialylated OS decreased; to our knowledge, this is the first report of a simultaneous increase of fucosylated OS and a decrease in sialylated OS. Interestingly, the present study did not detect any OS containing Neu5Gc, which suggest the OS profile of porcine milk more closely matches that of human milk than milk from other domestic animals, including bovine milk (29). Similar to what is reported for human milk, only a few OS contributed to the majority of the OS abundance in porcine milk. Human milk is considered unique as it contains type I OS [LNT, LNFP-I, lacto-N-fucopentaose II (LNFP-II), and LNDFH-I], none of which have ever been identified in bovine milk (4). Due to the proximity of elution times and the lack of MS-grade standards, it was not possible to distinguish among LNH/LNnH and LNT/LNnT isomers by Nano-LC Chip QTOF MS, but LNDFH-I was unambiguously detected in porcine milk and LNnH was successfully quantified using HPAE-PAD. Considering the lack of Neu5Gc, the contribution of just a few major OS to the overall OS present, the increase of fucosylation during lactation and the presence of LNDFH-I, evidence from this study suggests that porcine milk is closer in OS composition to human milk than previously established.

Quantification of OSs is generally limited to a few structures due to the paucity of commercial standards, which greatly impedes quantification of the diverse set of compounds that can be identified by Nano-LC Chip–Q-TOF MS. In the present study, eight neutral and three acidic OSs, which are found in human and/or bovine milk, were accurately quantified. The general trends in quantified OS match with observations of the global profiling by Nano-LC Chip–QToF MS, where sialylated OS decreased, and fucosylated OS increased, in abundance during lactation. The higher abundance of fucosylated versus sialylated OS structures places porcine milk OS (albeit in lower concentrations) as structurally closer to OS profiles found in human milk, rather than bovine milk. Additionally, the conspicuous absence of Neu5Gc-containing OS and the simultaneous presence of typical human milk OS, such as LNDFH-I, places porcine milk far closer to human milk than previously thought. Although porcine milk appears to closely resemble human milk composition, as stated above, there are some characteristics that tend to align more closely with bovine milk, namely individual OS concentrations in porcine milk tended to be lower than those found in human milk (30, 31) and rather similar to those found in bovine milk (32–34) with total OS concentrations decreasing throughout lactation.

Sialic acid is present in mammals’ biological fluids and tissues, contributing to the formation of complex structures, such as glycolipids or glycoproteins. Only 5% of the total SA typically exists in the free or unbound form (29). Following a similar trend to overall OSs, the total and free SA concentrations of porcine milk were lower than what has been described for human milk, yet similar to that found in bovine milk (15 mg/L) (35). However, it is important to note that there are relatively few studies that have evaluated SA concentrations of bovine milk, and variability exists in factors, including maternal breed (wide range of concentrations ranging from 15 to 500 mg/L) and methodologies employed for quantification (e.g., colorimetric vs. HPLC). Concentrations of free SA quantified in the present study appears high (i.e., free SA represented 33% of total quantified SA on day 0), though this ratio rapidly changed over the course of lactation, with free SA accounting for only 7.4% of total SA at the end of lactation. This dynamic in the form of SA throughout lactation suggests that porcine milk more closely matches human than bovine milk, which typically contain roughly 5% of the total SA acid as the free form. Notably, our results were lower than those described for human colostrum (35 and 1500 mg/L for free and total, respectively) (36, 37) and bovine milk (15–500 mg/L of total SA) (35, 38). Additionally, the ratio of free-to-bound SA appeared to continually decrease throughout lactation, suggesting that more SA was being incorporated into other compounds. The majority of SA present in the body, most notably the brain, is in a bound form (13); thus, the increase in bound SA in milk is of interest in understanding how this might confer benefits in neurodevelopment.

At the first stages of life, enzymes involved in SA synthesis and incorporation into other structures are not mature or active. In quantitative terms, this developmental insufficiency may increase the dietary SA requirement to maintain biological processes and ensure optimal development. As infant enzyme systems supporting SA production mature throughout the lactation period, their dietary requirements for free SA decrease, hence the biological basis for milk SA to decrease throughout lactation (35–37). The values for free SA reported in our study are in agreement with the literature, where a decrease was observed throughout lactation. Interestingly, our results also show an increase of total SA and sialylated structures over lactation, which was not previously reported. Taken together, these results might suggest that as lactation progresses, SA is incorporated into different structures rather than remaining free in the milk.

The present work expands on previous work in analysis of porcine OS by characterizing additional novel OS present in porcine milk, while also quantifying changes in SA across lactation. Moreover, this study suggests that although porcine milk OS concentration and the number of structures is lower than human milk, the OS profile appears to be closer to human milk rather than to bovine milk. Much of the similarities are based on the increased proportion of fucosylated OS during lactation and the ability of sows to synthetize OS commonly present in human milk. These findings support the use of pigs as an ideal model for studies on human nutrition, not only because of the striking similarities in intestinal, immune system, and brain development but also due to their similarities in milk OS composition.

## Author Contributions

RD, CG, SJ, LA, AM, BB, and MC were involved in project conceptualization. CG, SJ, LA, and AM were involved in daily project operations and data collection. JS and DB were responsible for analytical procedures. All authors were involved in data analysis, interpretation, and have reviewed and approved this manuscript.

## Conflict of Interest Statement

BB and MC are employees of Mead Johnson Pediatric Nutrition Institute. The remaining authors declare that the research was conducted in the absence of any commercial or financial relationships that could be construed as a potential conflict of interest.
